# Myomas and Adenomyosis: Impact on Reproductive Outcome

**DOI:** 10.1155/2017/5926470

**Published:** 2017-11-06

**Authors:** Nikos F. Vlahos, Theodoros D. Theodoridis, George A. Partsinevelos

**Affiliations:** ^1^2nd Department of Obstetrics and Gynecology, Aretaieion Hospital, National and Kapodistrian University of Athens, School of Medicine, 76 Vasilissis Sofias Av., 11528 Athens, Greece; ^2^1st Department of Obstetrics and Gynecology, Papageorgiou General Hospital, Aristotle University of Thessaloniki, Faculty of Health Sciences, School of Medicine, Ring Road, Municipality of Pavlos Melas, Area of N. Efkarpia, 56403 Thessaloniki, Greece; ^3^Assisted Reproduction-IVF Unit, MITERA Hospital, 6 Erithrou Stavrou Str., Marousi, 15123 Athens, Greece

## Abstract

Among uterine structural abnormalities, myomas and adenomyosis represent two distinct, though frequently coexistent entities, with a remarkable prevalence in women of reproductive age. Various mechanisms have been proposed to explain the impact of each of them on reproductive outcome. In respect to myomas, current evidence implies that submucosal ones have an adverse effect on conception and early pregnancy. A similar effect yet is not quite clear and has been suggested for intramural myomas. Still, it seems reasonable that intramural myomas greater than 4 cm in diameter may negatively impair reproductive outcome. On the contrary, subserosal myomas do not seem to have a significant impact, if any, on reproduction. The presence of submucosal and/or large intramural myomas has also been linked to adverse pregnancy outcomes. In particular increased risk for miscarriage, fetal malpresentation, placenta previa, preterm birth, placenta abruption, postpartum hemorrhage, and cesarean section has been reported. With regard to adenomyosis, besides the tentative coexistence of adenomyosis and infertility, to date a causal relationship among these conditions has not been fully confirmed. Preterm birth and preterm premature rupture of membranes, uterine rupture, postpartum hemorrhage due to uterine atony, and ectopic pregnancy have all been reported in association with adenomyosis. Further research on the impact of adenomyosis on reproductive outcome is welcome.

## 1. Introduction

Embryo implantation into the endometrial cavity has been long believed to be mainly driven by endometrial receptivity and to a lower extent by the embryo itself. In this context, impaired endometrial receptivity accounts for two-thirds, whereas embryo quality, in terms of both morphology under the microscope and genetic composition, accounts for one-third of implantation failures [1, 2]. Therefore the role of the endometrium in adverse reproductive outcome should not be disregarded. In this respect, endocrine disorders, inherited and acquired thrombophilias, immunologic abnormalities, and chronic inflammation may be responsible for reduced endometrial receptivity. Structural abnormalities, either congenital such as Mullerian anomalies or acquired ones, such as endometrial polyps, intrauterine adhesions, myomas, and adenomyosis, may compromise embryo implantation following both natural conception and assisted reproduction technologies.

Besides an adverse impact on implantation, both myomas and adenomyosis may interfere by various means throughout the duration of pregnancy and affect the obstetrical outcome [3–11].

## 2. Uterine Myomas

Uterine myomas, also called leiomyomata, fibroids, fibromyomas, leiomyofibromas, and fibroleiomyomas, are the most common benign uterine tumors. Evaluation by ultrasound reveals the incidence of fibroids as high as 60% by age of 35 years in African-American women and 40% in Caucasian women. The incidence increases to 80% and 70% by age 50 of years, respectively [12]. Thereby, race along with age represents risk factors for myoma development. Interestingly, race is associated with myoma growth rate, given that women of African descent hold a relatively constant rate throughout reproductive life, whereas in Caucasian women myomas keep up a faster growth rate until 35 and a slower one after the age of 45 [13]. Early menarche, nulliparity, caffeine, and alcohol consumption, obesity, and high blood pressure have all been found to increase the risk, whereas smoking, possibly implicated in relative alteration in estrogen metabolism, has been shown to decrease the risk of developing fibroids [14–21].

The pathogenesis of myomas is considered multifactorial. A somatic mutation in a single smooth muscle cell of the uterus is the triggering event, which explains the monoclonal origin of these tumors [14]. However, genetic and epigenetic factors, including steroid hormones, growth factors, cytokines, and chemokines, are also implicated in the development and growth of myomas [20, 22]. Although initially significant attention had been paid to estrogens, nowadays, progesterone and its receptors (PR-A and PR-B) are believed to play a key role in myoma growth, modulating the expression of growth factor signaling proteins and, among others, regulating genes associated with proliferation, apoptosis, and differentiation [14, 20].

Anatomically, myomas are monoclonal tumors expanding, as they grow, between normal myometrial cells creating a pseudocapsule, which consists a fibro-neurovascular bundle, which surrounds the fibroid and separates it from healthy myometrium [23]. Basically, thickened collagen fibers and blood vessels form a vascular ring, which has been described as the “ring of fire” by color Doppler, whereas by conventional grey scale ultrasonography forms a hyperechogenic ring around the myoma [23, 24]. Accumulating evidence supports the importance of this pseudocapsule in secreting, neurotransmitters, and neuropeptides, such as substance P (SP) and vasoactive intestinal peptide (VIP) as well as other molecules all of which are implicated in wound healing [25–27].

**Figure 1:**
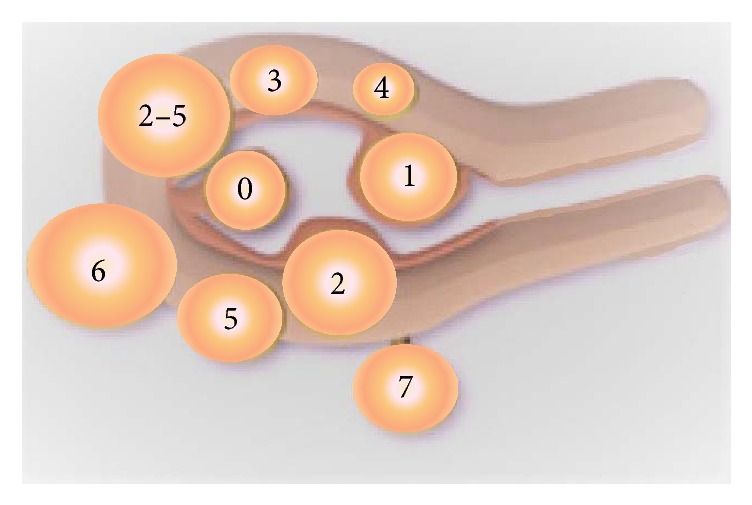
FIGO classification of myomas. FIGO classification system of myomas introduced by Munro and colleagues in 2011 [31] is based on the relationship of the fibroid with the uterine wall. According to this classification, type 0 to type 8, the last one representing fibroids, which cannot otherwise be classified, have been proposed, whereas for a subset of fibroids, two numbers may be applicable, the first one referring to the relationship with the endometrium and the second one with the perimetrium. This possibility can indirectly imply the size of a myoma, which, for instance, extends throughout the uterine wall protruding into the uterine cavity and concurrently distorts the outline of the uterus (type 2–5). Type 0: pedunculated intracavitary. Type 1 submucosal < 50% intramural. Type 2: submucosal ≥ 50% intramural. Type 3: entirely intramural, contacting the endometrium. Type 5: subserosal ≥ 50% intramural. Type 6: subserosal < 50% intramural. Type 7: subserosal pedunculated. Type 8 (not shown in the figure): others, that is, cervical, originating from the round ligament or parasitic.

Various systems have been proposed so far to describe myomas. Still, none of them takes into account all the parameters, which figure out the heterogeneity of these tumors. Traditionally, based on their location in relationship to the endometrial cavity, myomas are classified as submucosal, intramural, or subserosal [28–30]. The FIGO classification, introduced by Munro and colleagues in 2011, is based on the relationship of the fibroid with the uterine wall [31]. According to this classification, nine types of myomas have been described, from type 0 to type 8, the last one representing fibroids, which cannot otherwise be classified. For a subset of fibroids, two numbers may be applicable, the first one referring to the relationship with the endometrium and the second one with the perimetrium. This possibility can indirectly imply the size of a myoma, which for instance extend throughout the uterine wall protruding into the uterine cavity and concurrently distort the outline of the uterus (types 2–5) (Figure 1). Still, the size, the number, and the exact location the fibroids in relationship to the tubal ostium or the cervix are not taken into account into the classification.

Myomas are often asymptomatic and are diagnosed in routine ultrasound scan performed for other indications. Symptomatic myomas are associated with abnormal uterine bleeding (menorrhagia and/or metrorrhagia) pelvic pain due to myoma degeneration or torsion of a pedunculated myoma and pressure to adjacent organs, such as the bladder (urgency, frequency, or incontinence), ureters (hydronephrosis), pelvic veins (discomfort and pelvic pain), and rectum (constipation and tenesmus) [32–34].

Myomas can also have an adverse effect on reproductive outcome either by impairing fertility or by complicating the course of and the completion of a pregnancy.

### 2.1. Myomas and Infertility

Although myomas are present in 5–10% of infertile women, they present as a sole cause of infertility only in 2-3% [35], which means that hardly up to 60% of myomas may cause infertility [34].

Fertility impairment due to the presence of fibroids has been attributed to various mechanisms (Table 2).

Distortion of the uterine cavity, rendering the endometrial contour anomalous, may compromise implantation potential. Furthermore, sperm transport may be hampered by an enlarged and deformed fibroid uterus, whereas cervical displacement may hinder sperm passage into the cervical canal. The presence of myomas may also alter myometrial contractility, which in turn may compromise sperm progression into the female reproductive system. Alteration to the endometrial and myometrial blood supply due to underlying myomas may also interfere with both uterine contractility and implantation, whereas retained menstrual efflux due to a deformity of the uterine cavity may interfere with both sperm transport and implantation. Deviation or obstruction of the tubal ostia may compromise tubal patency and alteration of the tubo-ovarian anatomic relation may impede ovum collection from the fimbrial end following ovulation. Finally, a chronic inflammatory reaction to the adjacent endometrium, due to the presence of myomas, has been suggested to alter endometrial milieu [30, 34–41].

In fact, the closer the myoma to the endometrial cavity the worse the impact to the overlying endometrium as it has been shown that myomas lying close or in contact with the endometrial surface are associated with histologic alterations, which are known to impair implantation. In fact, endometrial atrophy, ulceration, elongation, and distortion of the endometrial glands, cystic glandular hyperplasia, polyposis, and endometrial venule ectasia have all been reported in the endometrium adjacent to the myoma. Interestingly, endometrial atrophy and ulceration are often evident even on the distal endometrium lying on the opposite uterine wall, probably due to a mechanical effect [30, 42–44].

In a study assessing the effect of uterine leiomyomas on the endometrium using molecular markers of endometrial receptivity, a decrease in HOX gene expression throughout the endometrium and not simply over a submucosal myoma was found. This observation implies that impairment of fertility may be attributed to a global effect and not simply a focal change of the endometrium overlying the myoma [45].

Mechanisms associated with fertility impairment in the presence of myomas frequently coexist, depending on their size, number, and location. However, in assisted reproduction technologies (in vitro fertilization), access of the ejaculated sperm to the cervical canal and sperm transport as well as tubal patency are irrelevant; therefore mechanisms that interfere with the implantation process may have a prominent role [30, 40].

Subserosal myomas, either sessile or pedunculated, distorting the outer uterine contour, do not seem to have a significant impact on fertility potential [36, 40, 46, 47]. Despite the fact that a recent systematic review and meta-analysis of controlled studies found that the presence of fibroids irrespective of their location significantly lowers implantation, clinical pregnancy, and ongoing pregnancy/live birth rates, when the analysis was restricted to subserosal myomas, no difference was observed for any of these endpoints. Therefore, subserosal myomas do not seem to affect fertility outcomes, and their removal does not confer any benefit [47].

The effect of intramural myomas on fertility is still somehow controversial, probably due to methodological limitations, but it has gained increased interest especially in the era of assisted reproduction. It was believed initially that myomas not protruding into the intrauterine cavity are not related to infertility; however, neither the number nor the size of the myomas was taken into account.

Several systematic reviews and meta-analyses have looked at this issue [36, 40, 46–48].

In 2001, Pritts failed to demonstrate an adverse effect of intramural fibroids on fertility in women undergoing assisted reproductive technologies (ART), thereby arguing against surgical intervention [46]. However, four years later, in 2005, Benecke and colleagues reported a negative impact of intramural fibroids on pregnancy rate in ART cycles [40]. In line with Benecke and colleagues, Somigliana and colleagues in an updated meta-analysis in 2007 found an adverse effect of intramural fibroids on ART outcome, in terms of clinical pregnancy and live birth rates [36, 49]. In this context, a systematic review and meta-analysis performed by Pritts and colleagues in 2009 demonstrated that intramural fibroids were associated with decreased implantation, clinical pregnancy, and ongoing pregnancy/live birth rates and higher miscarriage rates [47]. When only prospective studies were evaluated in this meta-analysis, all the aforementioned endpoints, but clinical pregnancy rates, were statistically significant. Finally, when only studies using hysteroscopy to evaluate the intrauterine cavity were assessed, the only significant impact of myomas was documented on implantation rates. It is noteworthy that this meta-analysis included both women undergoing assisted reproduction by any means (in vitro fertilization [IVF], intracytoplasmic sperm injection [ICSI], egg donation and/or embryo recipient program, and intrauterine insemination) and women attempting spontaneous conception. Subsequently, a systematic review and meta-analysis, Sunkara and colleagues focused on the effect of intramural myomas on fertility. They examined only intramural fibroids in respect to the outcome of in IVF [48]. They noticed that intramural fibroids, which by definition did not distort endometrial cavity, were associated with lower clinical pregnancy and live birth rates. However, when they focused their analysis on prospective studies only, they documented an adverse effect solely to live birth rates.

It is obvious that the exact location of an intramural fibroid may also determine its impact on fertility potential; that is to say, a fibroid lying on the cornual end or near the cervix may compromise sperm migration and thus fertilization.

Several efforts have been made to relate the size of an intramural fibroid with reproductive outcome. A cut-off of 2.85 to 7 cm for maximum myoma diameter has been assessed in the literature so far, yielding opposing results [50–54]. Although a recent retrospective cohort study suggested that intramural fibroids greater than 2.85 cm in diameter may negatively affect delivery rates in women subjected to IVF/ICSI treatment, accumulating evidence suggests that a diameter above 4 cm should be probably considered clinically significant from a reproductive aspect [52, 55].

In respect to submucosal myomas, the literature is quite clear. Current evidence highlights their detrimental effect on fertility. The FIGO and the ESGE classifications describe three types of submucosal myomas. Types 0, 1, and 2, of FIGO correspond to types 0, I, and II of ESGE and represent myomas being pedunculated and thus protruding entirely into the intrauterine cavity, sessile with less than 50% myometrial extension and sessile with more than 50% myometrial extension, respectively [56].

Current evidence based on available systematic reviews and meta-analyses, which have looked at the effect of submucosal myomas on fertility [36, 37, 40, 46, 47], all agree that submucosal myomas exert a detrimental effect on reproductive outcome. In a systematic review and meta-analysis conducted by Pritts, in women undergoing IVF, the presence of submucosal fibroids was associated with lower implantation (RR 0.28; CI 0.10–0.72) and pregnancy rates (RR 0.30; 95% CI 0.13–0.70) as compared with infertile controls devoid of fibroids. Surgical removal of these fibroids resulted in increased pregnancy rates (RR 1.72; 95% CI 1.13–2.58) and restored live birth rates (RR 0.98; 95% CI 0.45–2.41) [46]. A year later, Donnez and Jadoul reviewed the literature and ended up with a conclusion that although clear evidence is lacking, it seems reasonable that myomas distorting intrauterine cavity impair implantation and pregnancy rates in ART cycles [37]. In 2005, Benecke and colleagues again reinforced the aspect of a detrimental effect of submucosal fibroids on pregnancy rates in women subjected to ART [40] and in 2007, Somigliana and colleagues reported an adverse effect of submucosal myomas on ART outcome in terms of clinical pregnancy (RR 0.3; 95% CI 0.1–0.7) and live birth rates (RR 0.3; 95% CI 0.1–0.8) [36]. This was also the case in the systematic review and meta-analysis performed by Pritts and colleagues in 2009, in an unselected population undergoing assisted reproduction methods or even natural conception attempts. They found that submucosal fibroids were associated with a significant decrease in implantation (RR 0.283; 95% CI 0.123–0.649), clinical pregnancy (RR 0.363; 95% CI 0.179–0.737), ongoing pregnancy/live birth rates (RR 0.318; 95% CI 0.119–0.850), and higher miscarriage rates (RR 1.678; 95% CI 1.373–2.051) [47].

### 2.2. Myomas and Pregnancy Outcome

The prevalence of myomas during pregnancy has been reported to be as high as 12% (range 3–12%) [57–59]. Contrary to the traditional belief that myomas tend to grow in the course of pregnancy as a result of the high inherent estrogen levels, there is currently a wealth of evidence demonstrating that their size does not significantly increase and often becomes even smaller during pregnancy [10, 60–65].

Pain is the most common symptom associated with the presence of fibroids in the pregnant woman [66]. Although pain had been initially attributed to the tentative enlargement of fibroids, subsequent studies could not confirm such a firm relationship [66]. Pain should be probably attributed to prostaglandin release from fibroid degeneration, given the efficacious analgesic effect provided by nonsteroidal anti-inflammatory drugs [60].

Fibroids during pregnancy have been linked to adverse pregnancy outcomes. In fact, an increased risk of obstetric complications, such as miscarriage, fetal malpresentation, (primarily breech), placenta previa, preterm birth, placenta abruption, postpartum hemorrhage, and cesarean section in women carrying submucosal and/or large intramural fibroids, has been reported [57, 60].

Surgical treatment of uterine myomas, irrespective of the route of the approach, results in “scarred uterus,” which has been associated with increased probability of uterine rupture during subsequent pregnancy. It seems that the more the myoma nodule imbeds into the myometrium the higher the risk of uterine rupture. The risk is also increased in case of uterine perforation during hysteroscopy. Recent evidence also suggests that failure to identify and preserve fibro-neurovascular pseudocapsule during myomectomy may impair proper wound healing predisposing in uterine rupture [25–27]. In line with advice given following cesarean section, plans for future conception should be postponed, for six months after myomectomy. Nonetheless, some physicians recommend as long as a year of protected sexual intercourse following myomectomy [67]. Although, in respect to the mode of delivery in case of “scarred uterus,” no clear evidence exists, the depth of the uterine wall myoma occupied should not be ignored in decision-making among normal vaginal delivery and elective cesarean section [67, 68].

### 2.3. Treatment of Myomas from the Fertility Aspect

In general, there is a variety of surgical and medical options for the treatment of myomas. For the past several years minimal invasive approaches such as hysteroscopy and laparoscopy have gained popularity, whereas novel alternative minimal invasive methods, such as uterine artery embolization and noninvasive techniques, such as high frequency magnetic resonance-guided focused ultrasound surgery (MRgFUS), have also been used.

Surgical intervention is mainly determined by the type and the number of myoma. Submucosal myomas are optimally treated hysteroscopically using either mechanical instruments (scissors and mechanical “cold” loops), electrocautery (thermal loops and vaporizing electrodes), laser fibers (“touch” and “nontouch” technique) [69, 70], or intrauterine morcellation [68]. Although “resectoscopic slicing” of the myoma with the use of electrical energy is the more popular and widely applied technique, it has been blamed that it can inevitably damage the surrounding healthy myometrium, mainly in type 1 or 2 according to FIGO classification myoma resection, due to the poorly defined intermyoma-myometrium cleavage plane. Therefore, from the fertility aspect, the superiority of “cold loop” myomectomy, which combines both monopolar electrocautery for the excision of the intracavitary component and mechanical blunt dissection using mechanical loop for the enucleation of the intramural component of the submucosal fibroid, has been proposed [71]. Moving the loop on the reference plane under direct visual control and minimizing inadvertent electrosurgical damage, either direct through the monopolar loop or indirect through the thermal effect, respect of the surrounding healthy myometrium is ensured. Thus, future chances of conception are enhanced and potential complications of the “scarred uterus” during pregnancy are kept to a minimum [68].

Large sessile submucosal myomas extending >50% into the myometrium may require a two-step approach. During the first step, resection of the protruding part of the myoma allows the surrounding myometrium to contract and push the remaining further into the cavity. At the later time, complete resection of the residual intramural part, which has now migrated towards the intrauterine cavity, during a second-step hysteroscopy approach is possible [31].

Given that both FIGO and ESGE classifications do not take into account the size, the topography, and the extension of the base of the submucosal myoma with respect to uterine wall, Lasmar and colleagues in 2005 proposed a presurgical classification system including these parameters along with the extent of the penetration of the nodule into the myometrium for the assessment of the viability of hysteroscopic treatment. A score of 0 to 9 is applied, assigning submucosal myomas in three groups. Group I (score 0–4) implies low complexity hysteroscopic myomectomy, and Group II (score 5-6) is suggestive of a complex hysteroscopic myomectomy and advises either preparing with GnRH-analogue or two-stage surgery, whereas Group III (score 7–9) indicates submucosal myomas, which are not suitable for the hysteroscopic approach (Table 1) [72, 73].

The use of GnRH-agonists before surgery may be beneficial in case of hysteroscopic resection of large submucosal myomas. In fact, this medication is efficient in decreasing myomas' size, endometrial thickness, and vascularization as well as minimizing distending medium intravasation and thus fluid overload [21]. Furthermore, even if intravasation occurs, GnRH-agonists may preempt sex steroid-related impact on the Na+/K+-ATPase pump, thus eliminating the effect of hyponatremic encephalopathy, the latter having been recognized as a potential fatal complication of minimal invasive uterine surgery [74]. Restoration of iron deficiency anemia with medically induced amenorrhea and scheduling operative hysteroscopy at any time instead of awaiting the follicular phase are also benefits of GnRH-agonist presurgical treatment [21, 68]. However, up to now, there is no consensus regarding the indications and the duration of treatment with GnRH-agonists prior to hysteroscopic resection. Others however argue that increased cost, medication's side effects, high recurrence rate, and the “sinking” phenomenon meaning the difficulty in operating on the myoma due to the increased distention of the endometrial cavity as a result of pharmaceutical menopause do not justify the routine use of GnRH-agonists [68]. A rational approach would be the reservation of GnRH-agonist for pretreatment only for large (>3 cm) types 1 and 2 according to FIGO classification submucosal myomas, especially when anemia due to anomalous uterine bleeding complicates their presence. In this context, selective progesterone receptor modulators (SPRMs), such as ulipristal acetate, have been proposed for preoperative treatment. SPRMs in four three-month treatment course have been also proposed for women suffering from symptomatic fibroids, who wish to preserve their fertility in the future but unwilling to get pregnant at that moment. One to three-month treatment course with SPRMS have been recommended before IVF for women carrying intramural myomas or submucosal myomas that do not significantly distort the intrauterine cavity in order to improve implantation rates [21].

Laparoscopy, open abdominal surgery and combined laparoscopy and laparotomy (laparoscopic-assisted myomectomy) are indicated for intramural and subserosal myomas [75]. Furthermore, a minority of submucosal myomas judged by Lasmar et al.'s presurgical classification system not candidates for hysteroscopic resection as well as large (>3 cm) type 2 submucosal myomas occupying the entire myometrium are better treated through laparoscopy [72, 76].

Laparoscopy is apparently preferred, when available, given the minimally invasive nature of this technique compared to the alternative operative options. In fact, shorter hospitalization and recovery period and less postoperative pain, fever, and anemia have been observed in laparoscopic compared to abdominal myomectomy [77]. In order to preserve the anatomical and functional integrity of the uterus, myomectomy should respect basic surgical principles which guide against inadvertent healthy tissue damage. As myoma pseudocapsule shares similarities with the prostate capsule, myomectomy in correspondence to prostatectomy should focus on meticulous dissection of the neurovascular bundle and avoidance of extensive electrocoagulation with high electrical power (>30 watts). Such a surgical approach that spares the pseudocapsule is described as intracapsular myomectomy and seems to be advantageous compared with the extracapsular one, in terms of blood loss, operational time, and proper hysterotomy wound healing. Keeping on this principle, postoperative deficits in uterine muscular contractility, which affect reproductive and sexual function, are minimized [23].

GnRH-agonists have been found to make myomas shrink via confluent nodular hyaline degeneration and hydropic degeneration necrosis [78]. Although these actions may benefit hysteroscopic myomectomy, they are not desirable in laparoscopic and/or abdominal myomectomy, as the cleavage plane between healthy myometrium and the pseudocapsule may be obscured, resulting in copious dissection of the myoma and increased operating time with potential inadvertent distortion of the pseudocapsule [79].

In laparoscopy, the possibility of facing a uterine sarcoma (leiomyosarcoma followed by endometrial stromal sarcoma and carcinosarcoma) misdiagnosed as myoma exists, with a prevalence that ranges from 0.00% to 0.49% [80], although the risk has been probably overestimated [21]. To eliminate the risk of inadvertent tissue spread during surgery, “in bag” myoma excision and morcellation have been proposed to avoid ethical and medicolegal issues in case of unexpected malignancy [81–83].

Postmyomectomy adhesions, either intra-abdominal or intrauterine ones, may evolve irrespective of the surgical approach (hysteroscopy, laparoscopy, open abdominal surgery, or laparoscopically assisted myomectomy). Intrauterine synechiae are mainly linked to the hysteroscopic approach, especially when excessive electrosurgery is applied [71], unintended damage of the healthy endometrium, and myometrium proximal to the myoma occurs, and multiple submucosal myomas are resected laying on opposing uterine walls [68, 84]. Various modalities have been evaluated in the reduction of intrauterine adhesion formation following hysteroscopic myomectomy. Although hormone therapy using estrogens, application of intrauterine nonhormonal devices, urinary bladder (foley) catheters, uterine balloon, amnion graft, auto-cross-linked hyaluronic acid gel or combined hyaluronic acid, and carboxymethyl cellulose have shown promising results, none of them has been validated in abolishing posthysteroscopy intrauterine synechiae development [68, 85]. Nevertheless, early second-look hysteroscopy performed one to three weeks after surgery has been advocated to serve in prevention as well as early identification and treatment of adhesions at a stage that they will most likely be mild or moderate [86].

Intra-abdominal adhesions most often result from open abdominal surgery much more frequently as compared to laparoscopy. Poor surgical performance lacking gentle tissue handling is known to predispose to peritoneal adhesion formation. In this respect, electrocoagulation for hemostasis should be kept to a minimum. Among factors studied, 4% icodextrin solution, auto-cross-linked hyaluronic acid, expanded polytetrafluoroethylene, oxidized regenerated cellulose and the combined hyaluronic acid, and carboxymethyl cellulose have been shown to reduce postoperative adhesion development. However, there is no conclusive evidence on the relative effectiveness of these interventions [87–91].

Apart from surgical and medical strategies, the alternative minimal invasive approach of uterine artery embolization and the noninvasive high frequency magnetic resonance-guided focused ultrasound surgery (MRgFUS), which have been recently applied in myoma treatment, have not been adequately studied in cases, where fertility preservation is desired. Pregnancies have been reported following the application of both techniques, yet evidence is scanty to draw firm conclusions for women interested in childbearing [21, 37, 92, 93]. At this time fibroid artery embolization is a relative contraindication for women that desire to retain their reproductive potential [94, 95].

## 3. Adenomyosis and Adenomyomas

Adenomyosis is a nonneoplastic benign uterine disorder, characterized by the invasion of endometrium into the myometrium. In fact, heterotopic endometrial glands and stroma are found within the uterine musculature, surrounded by hypertropic and hyperplastic myometrium [96]. Adenomyosis typically occupies a large proportion of the uterus in a diffuse pattern rendering it bulky, and it is described as diffuse (adenomyosis). In general, posterior uterine wall is predominantly affected [97]. When adenomyosis is confined, it may present as a nodule (adenomyoma) occasionally misdiagnosed as myoma.

Adenomyosis was initially believed to be closely related to endometriosis, both having endometrial origin. In fact, it was thought that these entities represent different phenotypes of the same disorder. Later on and for the great proportion of the twentieth century, adenomyosis and endometriosis were distinguished from one another, until recently, when they were reconsidered as alternative expressions of a common entity [98]. To this end, technological advances in tissue imaging and the significant progress in molecular biology have served their best [99].

Taking into account the histologic characteristics, the extent and the location of the disease, Grimbizis and colleagues gathered diverse descriptions published in literature and proposed a new classification into diffuse and focal adenomyosis, the latter subdivided into adenomyoma with mainly solid characteristics and cystic adenomyosis, mainly described by the presence of a single adenomyotic cyst [100]. The term juvenile cystic adenomyosis (JCA) is reserved for the variant of focal cystic adenomyosis, which is present in women younger than 30 years of age with a cystic lesion larger than 1 cm and severe dysmenorrhea [101]. Polypoid adenomyomas, which present as circumscribed masses bulging into the endometrial cavity and are further subdivided into typical and atypical ones, and other forms such as adenomyomas of the endocervical type and retroperitoneal adenomyomas are considered rather distinct classes of the disease [100, 102–105].

For many years, the diagnosis of adenomyosis was based on histopathologic examination of hysterectomy specimens. Radiological modalities (hysterosalpingography) and gynecologic endoscopy procedures (hysteroscopy) for direct inspection of the intrauterine cavity did not fulfill initial expectations. Nowadays, transvaginal ultrasonography (TVS) and magnetic resonance imaging (MRI) may assist in the diagnosis of either diffuse or focal adenomyosis with a sensitivity of 72% and 77% and a specificity of 81% and 89%, respectively [106]. Still, in a significant proportion of cases only histopathology can confirm diagnosis. Given that hysterectomy is not an acceptable option for women willing to preserve their fertility, introduction of directed myometrial biopsy under sonographic, hysteroscopic, or laparoscopic guidance has yielded promising results [107–110].

While one-third of women carrying adenomyosis are asymptomatic, key clinical manifestations of this disorder include menorrhagia and dysmenorrhea. Clinical examination often reveals an enlarged tender uterus, and women may complain of chronic pelvic pain [111, 112].

The true incidence of adenomyosis is unknown, as definite diagnosis is based on histopathologic examination, whereas imaging modalities have been inconsistently used for diagnosis in the literature [99]. However, about 20% of women are believed to suffer from this entity [113].

Coexistence of adenomyosis with other gynaecological disorders, such as myomas and endometriosis, has been well established [97, 114–117]. A study evaluating the prevalence of adenomyosis using MRI scans in women diagnosed with endometriosis as compared to two control groups, one without endometriosis, defined as control group, and another without endometriosis but with a partner considered hypofertile, defined as healthy control group, confirmed the presence of adenomyotic lesions, in 79% of the endometriosis group, 28% of the control group, and 9% in the healthy control group [97]. Interestingly, the prevalence of adenomyosis reached 90% in the subset of women with endometriosis less than 36 years of age. This study contrasts findings from a previous study, in which adenomyosis diagnosed with MRI was present in only 27% of women with endometriosis [117].

### 3.1. Adenomyosis and Infertility

Epidemiological data suggesting that an increased prevalence of adenomyosis in multiparous women [118, 119] during the second half of their reproductive period of life should be interpreted with caution. In fact, these findings come up from older studies looking for adenomyosis on hysterectomy specimens, whereas nowadays the diagnosis of adenomyosis is feasible using noninvasive approaches, such as MRI and ultrasonography, through which the prevalence seems significant even in younger childless women. Therefore, the hypothesis that nulliparity may have a protective effect for the development of adenomyosis per se or that adenomyosis may not have a negative impact on the course of pregnancy does not seem to be fully justified.

To date there is no definite proof regarding the possible association between adenomyosis and infertility. At a first glance, the increased incidence of the disease in hysterectomy specimens of multiparous women in their 4th and 5th decade of life, presumable turns away such a link [120].

However, a pioneer study in baboons confirmed the presence of adenomyosis and reported a strong causal relation between adenomyosis and life-long primary infertility, even when cases of coexisting endometriosis were excluded (odds ratio 20.6, 95% CI 2.7–897) [121].

Subsequent reports in humans may have also suggested such a relation; however, most of them are case series with a level of evidence not strong enough to draw firm conclusions [122, 123]. Furthermore, design flaws, that is, potential coexistence of endometriosis, methodology used for diagnosis, that is, imaging instead of the traditional gold standard histopathology on hysterectomy specimen or even the less invasive targeted biopsy, may have compromised the evidence coming up from these studies. Nevertheless, the introduction of MRI during the last two decades facilitated the research on the effect of adenomyosis on reproductive outcome. In fact, the identification of the junctional zone, extending between the endometrium and the inner myometrium, and the validation of the diagnostic criteria through this imaging technique allowed the relatively accurate noninvasive diagnosis of this condition [124, 125].

It is well known that sperm following ejaculation is both actively, via progressive motility, and passively, via uterine peristaltic activity, transported in a cervicofundal direction to the ipsilateral fallopian tube, which corresponds to the ovary, where ovulation takes place [126]. Myometrial activity in the nonpregnant uterus has been shown to originate from the junctional zone, the latter being altered in the case of adenomyosis. Thus, aberrant uterine contractility impairing rapid and sustained directed sperm transport has been proposed as a plausible mechanism of infertility attributed to adenomyosis [127].

However, during the peri-implantation period, myometrial activity should be kept to a minimum to expedite apposition, adhesion, and penetration of the embryonic pole of the blastocyst into the decidualized endometrium. Research focusing on myometrial contraction patterns during embryotransfer has shown lower implantation and pregnancy rates in higher frequency junctional zone uterine activity and* vice versa* [128–130]. Although, increased contractility has been found in endometriosis, still, in adenomyosis, evidence is inadequate to definitely consider abnormal myometrial activity during the peri-implantation period as an additional mechanism for reproductive failure [99].

Endometrial receptivity seems to be also impaired in adenomyosis. Endometrial stroma vascularization has been found to be unexpectedly increased in the secretory phase, probably deranging the endometrial milieu, thus negatively affecting implantation [131].

Alterations in the expression profile of cytokines and growth factors in the endometrium have been linked to adenomyosis-associated infertility. Factors that are increased in patients with adenomyosis compared to normal fertile women include hypoxia-inducible factor 1*α* (HIF-1*α*) and interleukins (IL-6, IL-8, IL-10) as well as IL-8 receptors CXCR1 and CXCR2, matrix metalloproteinases (MMP2 and MMP9), and vascular endothelial growth factor (VEGF), whereas factors being decreased include leukemia inhibiting factor (LIF), LIF receptor *α*, and IL-11 [95]. A significant decrease in the expression of HOXA-10 gene during the midluteal phase has been documented in women with adenomyosis [132]. HOXA-10 gene expression is considered a necessary component of endometrial receptivity and peaks during the implantation window; therefore the decreased expression found in adenomyosis, as well as its counterpart endometriosis, may, at least partly, explain the detrimental effect of the disease in fertility [133].

Increased expression of cytochrome P450 in the endometrium along with increased aromatase activity has been proposed as possible mechanisms negatively affecting implantation in women with adenomyosis [134, 135].

In fact, local conversion of androgens to estrogens results in a hyperestrogenic endometrial environment, which sustains the increased expression of the estrogen receptor *α* during the secretory phase, which should have normally declined under the effect of progesterone. The hyperestrogenic endometrial milieu along with the overexpression of estrogen receptors adversely affect the expression of cell-adhesion molecules, such as *β*3 integrins, which are deemed as key elements for the development of a receptive endometrium [95].

Table 2 summarizes the mechanisms proposed for fertility impairment due to the presence of adenomyosis.

Besides the rationale for the existence of a link between adenomyosis and infertility, to date a causal relationship between these conditions has not been fully confirmed [100]. On the other hand, reports of the incidence of adenomyosis in the infertile women entering an IVF/ICSI program are inconsistent, varying from 6.9% to 34.3% [11]. A recent systematic review and meta-analysis on the effect of adenomyosis on IVF outcome reinforced the aspect of a negative impact of this condition on reproductive outcome [11]. Clinical pregnancy rates in women with adenomyosis were 28% lower as compared to controls (RR 0.72; 95% CI, 0.55–0.95). It is noteworthy that no significant difference was seen when analysis was restricted to women undergoing a single IVF/ICSI cycle (RR 0.80; 95% CI, 0.53–1.20). Interestingly, coexistence of endometriosis did not alter these results. Similarly, implantation rates were 23% lower in the adenomyosis group (RR 0.77; 95% CI, 0.63–0.93) and live birth rates were 30% lower (RR 0.70; 95% CI, 0.56–0.87). The miscarriage rate per clinical pregnancy was also significantly increased in women with adenomyosis (RR 2.12; 95% CI, 1.20–3.75). The authors concluded that screening for adenomyosis in infertile women entering an IVF program is worthy and thus should be encouraged.

### 3.2. Adenomyosis and Pregnancy Outcome

Data concerning the association between adenomyosis and obstetrical outcome are scanty. An early study reported a prevalence of adenomyosis of 17.2% in women undergoing cesarean hysterectomy. The authors went their thoughts a long way assuming that the presence of adenomyosis could have impair gravid uterus functionality, thereby increasing pregnancy complications, such as postpartum hemorrhage, uterine atony, and uterine rupture [136].

A subsequent and more recent study found an increased risk for preterm birth and preterm premature rupture of membranes in association with adenomyosis [137]. Among the pathogenic processes having been proposed so far, the authors pointed at decidual chorioamniotic or systemic inflammation, as the possible underlying mechanism for adenomyosis-related preterm delivery.

A review of the literature regarding obstetric complications in association to adenomyosis revealed only 29 cases. In particular, uterine rupture, postpartum hemorrhage due to uterine atony, and ectopic pregnancy were reported in relation to adenomyosis in the gravid uterus [138].

To date, evidence is not strong enough to support that adenomyosis affects the risk of obstetrical outcomes.

### 3.3. Treatment of Adenomyosis from the Fertility Aspect

For women suffering from adenomyosis that have completed their family, total hysterectomy could be considered the gold standard approach for symptom relief. However, for a patient, who has a desire to preserve her reproductive function, various uterine-sparing surgical techniques have been proposed. For patients with focal disease and for selected cases of more diffuse adenomyosis, excision of the adenomyoma or cystectomy for cystic focal adenomyosis has been proposed [100]. Partial removal of the abnormal tissue or cytoreductive surgery is reserved for cases of diffuse adenomyosis with special attention to preserve a functional uterus [100]. Nonexcisional invasive treatments include laparoscopic (electrocoagulation, uterine artery ligation), hysteroscopic (ablation, transcervical resection), and other treatments, the latter including uterine artery embolization [139] and ablation with MRI-guided focused ultrasound surgery (MRIgFUS), thermoballoon, radiofrequency, or microwave [100].

Conservative medical approaches have also been applied to relieve symptoms and in women wishing to get pregnant. GnRH-analogues, aromatase inhibitors, the levonorgestrel-releasing intrauterine contraception device, a danazol intrauterine contraception device, and the continuous use of estrogen-progestin oral contraceptives are all included in available treatment options [113, 140–146].

## 4. Conclusions

Myomas and adenomyosis represent common benign uterine pathologies with a remarkable prevalence in women of reproductive age. Although these entities often coexist, their pathophysiology and clinical characteristics are distinct. However, both disorders have been repeatedly linked to infertility.

In the era of evidence based medicine, submucosal myomas, which by definition distort the intrauterine cavity, have been consistently linked to an adverse effect on reproductive outcome and should be removed. The evidence is also abundant for subserosal myomas, which do not seem to be associated with infertility and adverse pregnancy outcome. However, the impact of intramural myomas on reproduction potential is not clear enough. Contemporary evidence suggests a causal relationship between intramural myomas larger than 4 cm in diameter and infertility.

The presence of submucosal and/or large intramural myomas has also been linked to adverse pregnancy outcomes, such as increased risk for miscarriage, fetal malpresentation, placenta previa, preterm birth, placenta abruption, postpartum hemorrhage, and cesarean section.

In respect to adenomyosis, the utilization of magnetic resonance imaging and modern ultrasonography has provided adequate accuracy in the diagnosis of the disease abolishing the need for histopathologic confirmation. Despite the confirmed clinical association between adenomyosis and infertility, to date a causal relationship between these conditions has not been fully confirmed, although it has been repeatedly suggested. An association between obstetrical complications, such as preterm birth, preterm premature rupture of membranes, uterine rupture, postpartum hemorrhage, and ectopic pregnancy adenomyosis, has also been reported. Still, the precise role of adenomyosis on reproductive outcome is not well clarified.

## Figures and Tables

**Table 1:** STEP-w classification for myomas.

Points	Size (cm)	Topography	Extension of the base	Penetration	Lateral wall
0	≤2	Low	≤1/3	0%	+1 point
1	>2–5	Middle	>1/3–2/3	≤50%	
2	>5	Upper	>2/3	>50%	

According to STEP-w classification system of myomas proposed by Lasmar and colleagues in 2005 [72, 73], the size, the topography, the extension of the base of the submucosal myoma with respect to uterine wall, and the extent of the penetration of the nodule into the myometrium are taken into account in presurgical evaluation of the viability of hysteroscopic treatment. A score of 0 to 9 is applied, assigning submucosal myomas in three groups: Group I (score 0–4): low complexity hysteroscopic myomectomy; Group II (score 5-6): complex hysteroscopic myomectomy consider preparing with GnRH-analogue and/or two-stage surgery; Group III (score 7–9): recommend alternative nonhysteroscopic treatment.

**Table 2:** Mechanisms proposed for fertility impairment on the presence of myomas and adenomyosis.

	Mechanism
Myomas	(i) Distortion of the uterine cavity rendering the endometrial contour anomalous may compromise implantation potential (ii) An enlarged and deformed fibroid uterus may hamper sperm transport (iii) Cervical displacement may hinder sperm passage into the cervical canal (iv) Altered myometrial contractility may compromise sperm progression into the female reproductive system (v) Alteration to the endometrial and myometrial blood supply may interfere with both uterine contractility and implantation (vi) Retained menstrual efflux due to a deformity of the uterine cavity may interfere with both sperm transport and implantation (vii) Deviation or obstruction of the tubal ostia may compromise tubal patency (viii) Alteration of the tubo-ovarian anatomic relation may impede ovum collection from the fimbrial end following ovulation (ix) Chronic endometrial inflammation due to myomas lying adjacent to the endometrium may alter endometrial milieu (x) Histologic alterations attributed to myomas lying close or in contact to the endometrial surface may impair implantation (xi) Among molecular markers of endometrial receptivity, a decrease in HOX gene expression throughout the endometrium and not simply over a submucosal myoma may suggest that impairment of fertility may be attributed to a global effect and not simply a focal change over the myoma

Adenomyosis	(i) Aberrant uterine contractility, originating from the junctional zone, which is broadened in case of adenomyosis, may impair rapid and sustained directed sperm transport (ii) Abnormal myometrial activity during the peri-implantation period may hinder apposition, adhesion, and penetration of the embryonic pole of the blastocyst into the decidualized endometrium (iii) Increased endometrial stroma vascularization in the secretory phase may derange the endometrial milieu, thus negatively affecting implantation (iv) Alteration in the expression profile of cytokines and growth factors in the endometrium, such as increased expression of hypoxia-inducible factor 1*α* (HIF-1*α*) and interleukins (IL-6, IL-8, IL-10) as well as IL-8 receptors CXCR1 and CXCR2, matrix metalloproteinases (MMP2 and MMP9) and vascular endothelial growth factor (VEGF) and decreased expression of leukemia inhibiting factor (LIF), LIF receptor *α*, and IL-11 may be linked to adenomyosis-associated infertility (v) Decreased expression of HOXA-10 gene during the midluteal phase, which is considered a necessary component of endometrial receptivity and peaks during the implantation window, may negatively affect implantation (vi) Hyperestrogenic endometrial environment due to the increased expression of cytochrome P450 along with increased aromatase activity in the endometrium sustains the increased expression of the estrogen receptor *α* during the secretory phase. This in turn adversely affects cell-adhesion molecule expression, such as *β*3 integrins, which are deemed as key elements for the development of a receptive endometrium
